# The Impact of Hemp Shives Impregnated with Selected Plant Oils on Mechanical, Thermal, and Insulating Properties of Polyurethane Composite Foams

**DOI:** 10.3390/ma13214709

**Published:** 2020-10-22

**Authors:** Sylwia Członka, Anna Strąkowska, Agnė Kairytė

**Affiliations:** 1 Institute of Polymer & Dye Technology, Lodz University of Technology, 90-924 Lodz, Poland; anna.strakowska@p.lodz.pl; 2 Laboratory of Thermal Insulating Materials and Acoustics, Institute of Building Materials, Faculty of Civil Engineering, Vilnius Gediminas Technical University, Linkmenu st. 28, LT-08217 Vilnius, Lithuania; agne.kairyte@vgtu.lt

**Keywords:** polyurethanes, hemp shives, bio-filler, oil impregnation, mechanical properties

## Abstract

Polyurethane (PUR) foams reinforced with 2 wt.% hemp shives (HS) fillers were successfully synthesized. Three different types of HS fillers were evaluated—non-treated HS, HS impregnated with sunflower oil (SO) and HS impregnated with tung oil (TO). The impact of each type of HS fillers on cellular morphology, mechanical performances, thermal stability, and flame retardancy was evaluated. It has been shown that the addition of HS fillers improved the mechanical characteristics of PUR foams. Among all modified series, the greatest improvement was observed after the incorporation of non-treated HS filler—when compared with neat foams, the value of compressive strength increased by ~13%. Moreover, the incorporation of impregnated HS fillers resulted in the improvement of thermal stability and flame retardancy of PUR foams. For example, the addition of both types of impregnated HS fillers significantly decreased the value of heat peak release (pHRR), total smoke release (TSR), and limiting oxygen index (LOI). Moreover, the PUR foams containing impregnated fillers were characterized by improved hydrophobicity and limited water uptake. The obtained results confirmed that the modification of PUR foams with non-treated and impregnated HS fillers may be a successful approach in producing polymeric composites with improved properties.

## 1. Introduction

Recently, the synthesis and development of polyurethane (PUR) composites containing natural fillers have attracted increased attention in industry and academia [1,2,3]. The application of natural fillers as reinforcing materials in the production of PUR foams has both ecological and economic advantages. Among the natural materials, cellulosic compounds have significant advantages, mostly due to their low density, high stiffness, biodegradability, unlimited availability, and low price. Previous studies have shown that the incorporation of organic and inorganic materials into the polymer matrix may successfully improve the mechanical characteristics of PUR composites [4,5,6] (Table 1). For example, the basalt waste has been used as a reinforcing filler for the production of rigid PUR foams by Kurańska et al. [7]. Due to the incorporation of 3–40 wt.% of the powdered basalt filler, the resulting PUR composite foams were characterized by improved mechanical performances. Similar results have been reported by Paciorek-Sadowska et al. [8] in the case of PUR composite foams containing 30–60 wt.% rapeseed filler. PUR composites with increased apparent density and enhanced mechanical properties were produced. The improvement of mechanical and thermal performances was also observed after the incorporation of egg-shells [9]. The addition of 20 wt.% egg-shells resulted in a significant improvement of the abovementioned properties. Interesting results were presented by Olszewski et al. [3] in the case of PUR foams containing glass and sisal fibers—the flexural strength, impact strength, and hardness of materials have been improved by the addition of both kinds of fibers. The effect of waste sludge particles on the physical and mechanical properties of PUR foams was studied by Kairyte et al. [10]. The authors reported that the addition of 20 wt.% of the fillers results in the production of the PUR materials with improved characteristics, while the higher content of the filler slightly deteriorates the properties of the foams. Interesting results were presented by de Avila Delucis et al. [11] who synthesized PUR foams reinforced with different ratios (1, 5, and 10 wt.%) of forest-derivatives fillers, e.g., bark, pine trees needles, kraft lignin, and paper sludge. Among the modified samples, the most promising materials were PUR foams reinforced with 1 and 5 wt.% wood, which exhibited improved mechanical and hygroscopic performance.

Among different organic fillers, the chemical composition of hemp shives have great potential as sustainable reinforcements for novel polyurethane composite foams. The basic unit of hemp shives is composed of cellulose microfibrils, which are combined by an interphase mixture of different pectins, hemicellulose, and other low-molecular polysaccharides [29]. The hydrogen bonds between different chemical components provide stiffness and mechanical strength of hemp shives. For example, hemicellulose determines the thermal degradation and moisture absorption, while the lignin content determines the UV degradation of the hemp shives [30,31]. Hemp shives offer several advantages, such as sufficient reactive functional groups, high carbon content, compatibility with diverse industrial chemicals, good stability and mechanical properties due to the presence of aromatic rings, and good rheological and viscoelastic properties, making it a potential candidate to be used as reinforcing material in polymer composites.

Nevertheless, the application of cellulosic materials as reinforcement of polymeric composite materials presents some limitations. As for mechanical strength, chemical or physical treatment of cellulose surface may improve the mechanical and thermal properties of polymer composites [32,33,34]. In previous works, a chemical modification, such as acetylation [35], alkalization [36], benzoylation [37] of cellulosic compounds, has been reported. For example, Du et al. have reported an improvement of interfacial compatibility between polyimide matrix and wood fibers treated by 3-Aminopropyltriethoxysilane [38]. Such reinforced composites were characterized by improved abrasive and tensile properties. Alkali-treated coir fibers were developed by Valášek et al. [39]. Due to the improved interphase adhesive, epoxy composites reinforced with alkali-treated coir fibers exhibited improved mechanical properties. Similar results have been shown in the case of epoxy composites reinforced with palm fibers chemically treated with sodium hydroxide [40]. Improvement of wear characteristics and mechanical performances of composites was observed due to the addition of the fibers. Chemical treatments of oil palm fibers, such as latex coating, acetylation, or acrylonitrile grafting have been evaluated by Sreekala et al. [41]. The authors have shown that phenol formaldehyde composites containing modified fibers were characterized by better flexural characteristics and improved impact resistance.

Many previous works have studied the impact of natural fillers on the mechanical and thermal characteristics of polymeric composites; however, no studies have been devoted to the examination of the polyurethane foam composites reinforced with physically-treated hemp shives. Keeping in view the advantageous properties of hemp shives, it seems logical to use hemp shives as a reinforcing filler for new bio-based polyurethane composite foams. The preparation of novel materials from hemp shives products may improve the mechanical properties of the polyurethane materials as well as possibly solve the problem of their waste disposal. Therefore, the impact of hemp shives impregnated with sunflower oil and tung oil on morphological, mechanical, and thermal properties of polyurethane foam composites was examined.

## 2. Materials and Methods

### 2.1. Materials

PUR foams were synthesized using polyether polyol (Stapanpol PS-2352) and polymeric diphenylmethane diisocyanate (Purocyn B). As catalysts, Kosmos 75 and Kosmos 33 (potassium octoate and potassium acetate, respectively) were used. Silicone surfactant (Tegostab B8513) was used for stabilizing the foam’s structure and the mixture of pentane and cyclopentane (50:50 *v*/*v*%) was selected as a blowing agent in forming cellular structure. Hemp shives, sunflower oil, and tung oil were obtained from a local company.

### 2.2. Methods

#### 2.2.1. Impregnation of Hemp Shives (HS) with Sunflower Oil and Tung Oil

Hemp shives were milled and wetted with selected oil (sunflower/tung oil). The mixture was thoroughly mixed and poured into cups. Subsequently, the cups with the mixture were put into the vacuuming dish and the vacuuming process proceeded until 0.01 MPa of pressure was achieved. Then, the green handle of the vacuum dish was screwed and the vacuum was left for another 30 min. A total of 10 cycles were done for the mixtures and, after that, all mixtures were thermally treated at 70 °C for 24 h. After the thermal treatment, the mixtures were left to cool down at 23 ± 5 °C temperature and 50 ± 5% humidity conditions.

#### 2.2.2. Synthesis of PUR Foams

**Figure 1:**
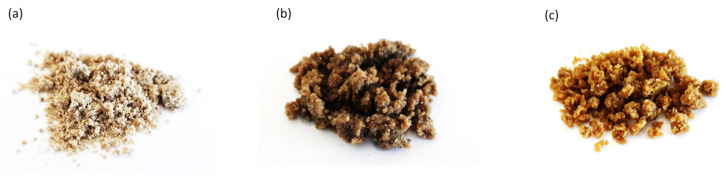
Hemp shives (HS) fillers used as reinforcing fillers: (**a**) non-treated hemp shives (HS), (**b**) hemp shives impregnated with sunflower oil (HS/SO), and (**c**) hemp shives impregnated with tung oil (HS/TO).

**Figure 2:**
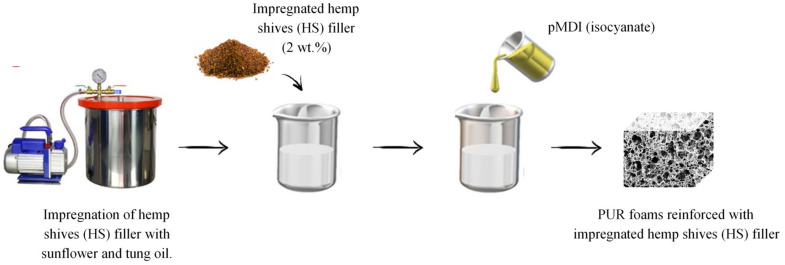
Synthesis of PUR foams reinforced with HS fillers.

PUR foams were produced by a one-shot method according to the procedure reported in the previous works. In brief, the synthesis of PUR foams modified with the addition of HS was as follows (Figure 1): To form a polyol premix, the calculated amounts of polyol (Stepanpol), catalysts (Kosmos 75 and Kosmos 33), blowing agent (the mixture of pentane and cyclopentane), and surfactant (Tegostab) were placed in a plastic cup and intensively mixed at 1500 rpm by a mechanical stirrer for 60 s. Then, the previously impregnated HS fillers were added to the cup and mixed for another 60 s to form a homogenous dispersion. A calculated amount of isocyanate (Purocyn) was added to the reaction mixture and thoroughly mixed for 10 s. The free rise PUR composite foam was left at room temperature for 24 h to provide complete curing of composites. A schematic procedure for the synthesis of PUR foams is shown in Figure 2. PUR foams were synthesized following the formulations presented in Table 2.

#### 2.2.3. Sample Characterization

The dynamic viscosity of polyol premixes was examined following ISO 2555 [42] using Viscometer DVII+. (Viscometer DVII+, Brookfield, Berlin, Germany). The cellular structure of PUR foams was evaluated using a scanning electron microscope using JSM-5500 LV (JEOL JSM 5500 LV, JEOL Ltd., Peabody, MA, USA). The cell sizes of PUR foams was determined by ImageJ software (Java 1.8.0, Media Cybernetics Inc., Rockville, MD, USA). The average pore diameters, pore size distribution, and the closed-cell content were identified based on SEM micrographs using the binarization threshold—an average of 400 individual measurements was reported. The apparent density of PUR foams was calculated as the ratio between the weight and volume of the samples according to ISO 845. The number of closed-cells was evaluated according to the ISO 4590 standard. Thermal conductivity (λ) of PUR foams was measured at 25 °C by using LaserComp 50. The mechanical performances of PUR foams were performed using a Zwick Z100 Testing Machine (Zwick/Roell Group, Germany). Compressive strength was examined parallel to the foam rise direction according to the ISO 844 [43] standard. Flexural and impact strength of PUR foams were evaluated according to the ISO 178 [44] and ISO 180 [45] standards. The dynamic–mechanical characteristic (DMA) was performed using an ARES rheometer (ARES, TA Instruments, New Castle, DE, USA) under the selected parameters (applied deformation of 0.1% and a frequency of 1 Hz, temperature range of 0–250 °C). The thermogravimetric analysis (TGA) test was performed in the function of temperature (0–600 °C) using an STA 449 F1 Jupiter Analyzer (Netzsch Group, Selb, Germany). The fire behavior of PUR foams was evaluated using a cone calorimeter apparatus according to ISO 5660 in S.Z.T.K. “TAPS”—Maciej Kowalski Company (Lodz, Poland).

## 3. Results and Discussion

### 3.1. Topography and an Average Size of HS Fillers

**Figure 3:**
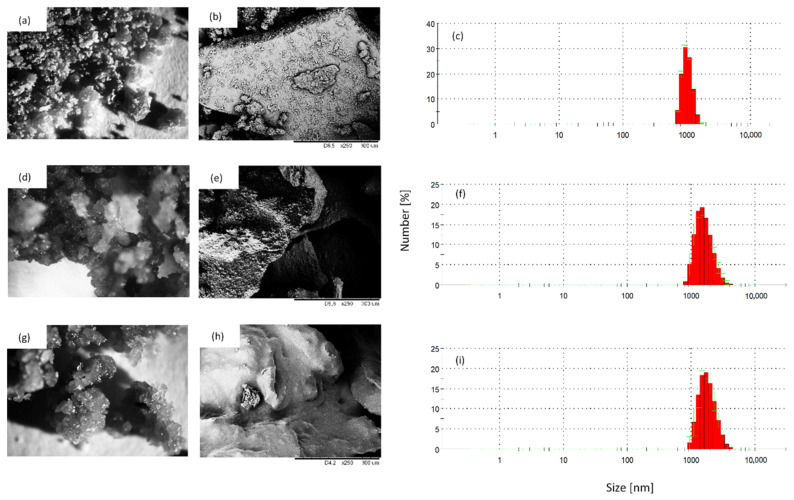
Topography and particle size of (**a**–**c**)—HS filler, (**d**–**f**)—HS/SO filler and (**g**–**i**)—HS/TO filler.

The external morphology of HS, and impregnated HS fillers (HS/SO and HS/TO), is presented in Figure 3. Comparing non-treated HS and impregnated HS fillers, it is clear that the oil impregnation affects an external morphology of the filler. After the impregnation with sunflower and tung oils, the fillers possess a similar structure; however, the particles tend to agglomerate, forming the bigger clusters of filler particles. The size of HS particles ranges from 400 to 800 nm, while, after the impregnation with sunflower oil, the average size of particles increases and it ranges from 3 to 5 µm. A similar relationship is observed for HS impregnated with tung oil—the average diameter ranges from 3 nm to 6 µm. Moreover, the addition of HS fillers increases the viscosity of the PUR systems (Table 3). Among all modified systems, the greatest viscosity is observed for PUR systems containing impregnated HS fillers. The viscosity increases rapidly from 840 (for neat PUR system) to 1800 and 2200 mPa·s after the addition of HS fillers impregnated with sunflower and tung oil, respectively. This result is not surprising, considering a high tendency of the filler to agglomeration and formation of coarse domains, as presented in Figure 3.

### 3.2. Reactivity of PUR Foam Formulations

The reactivity of PUR systems was investigated by measuring the start time, free rise time, and tack-free time during the foaming process (Table 3). Incorporation of HS, HS/SO, and HS/TO decreased the reactivity of the PUR systems. When compared to neat PUR_0, the foaming reaction rate was lower as HS fillers were introduced, probably due to the steric hindrance effects of hydroxyl groups (-OH) of HS filler. On the other hand, the presence of hydroxyl groups of HS filler can affect the proper stoichiometry of PUR synthesis due to the reaction between hydroxyl groups of HS filler and isocyanate groups. Consequently, the higher number of isocyanate groups is consumed and the reduced amount of carbon dioxide is produced, slowing down the foaming behavior of PUR systems. The extended processing times may be also connected with increased viscosity of the PUR dispersion containing HS fillers, which affect the expansion of PUR systems, extending the free rise time of PUR foams. Comparing PUR systems containing HS fillers, the highest values of processing times are observed for PUR_HS/TO, which may be connected with the higher viscosity of PUR systems containing HS/TO and the presence of bigger aggregates of HS particles. Similar results have been also found in previous works [46].

### 3.3. Morphology, Apparent Density and Thermal Conductivity of PUR Foams

**Figure 4:**
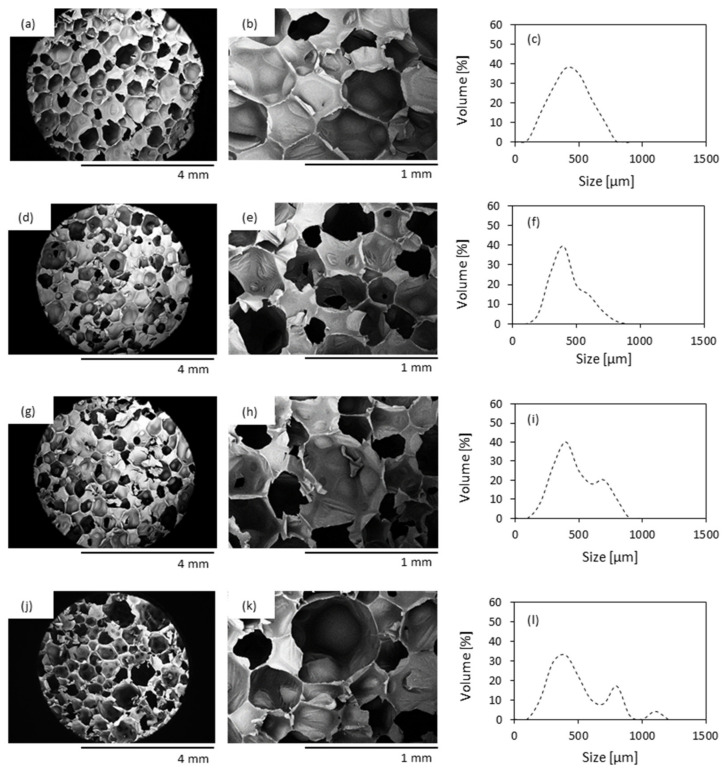
Optical image, SEM image and cell size distribution of (**a**–**c**) PUR_0, (**d**–**f**) PUR_HS, (**g**–**i**) PUR_HS/SO and (**j**–**l**) PUR_HS/TO.

Figure 4 presents the SEM images of PUR foams containing HS fillers without and with oil impregnation. The morphology of neat PUR_0 is smooth and regular. With the incorporation of HS fillers, the cellular shape is more irregular with the formation of a higher number of open cells. It can be seen that, with the incorporation of impregnated HS/SO and HS/TO, some agglomerates of the fillers are visible in the cell struts. The alteration in cellular morphology may be connected with increased viscosity of PUR systems containing HS fillers. As a result, the formation and expansion of air bubbles are hindered, which results in the creation of a more heterogeneous structure of PUR foams. When compared to neat PUR_0, the overall shape of cells becomes more irregular after the incorporation of HS fillers. All series of modified PUR foams possess a poor structure with a higher number of open cells. This effect is more prominent in the case of PUR foams containing both types of impregnated HS fillers. Similar dependence was also observed in previous works and was connected with the attachment of the filler particles to the cell walls leading to the rupturing and collapsing of foam’s cells and ultimately to the weakening of the modified foam’s structure [47]. The addition of HS fillers affects average cell size and this effect is more prominent after the incorporation of impregnated HS fillers (Figure 4). In general, neat PUR_0 possess an average cell size of 450 µm. The addition of HS fillers results in the formation of more inhomogeneous PUR foams with an average size in the range of 380–620 µm for each series of modified foams. Moreover, the addition of HS fillers decreases the content of closed-cell—the value decreases from 91.4% (for neat PUR_0) to 89.2, 88.6, and 85.6% for PUR_HS, PUR_HS/SO, and PUR_HS/TO, respectively. Previous studies have shown that the opening of cells due to the incorporation of organic filler may be connected with poor interphase adhesion between polyurethane matrix and filler surface, which results in disruption of the foaming process and more defective morphology of modified PUR foams [48]. Such an explanation may be found in our study as well. According to SEM results, some particles of HS fillers are localized in empty pores and they are not completely built in the foams’ struts. This confirms the poor compatibility between the filler and PUR matrix, leading to the cell collapsing and formation of PUR foams with open-pore structure [47]. Moreover, with the addition of HS fillers, the gas may form additional nucleation sites on the surface of HS filler particles, providing heterogeneous centers for the formation of air bubbles, which in turn increase the cell number of the PUR foam structure. As reported previously, the viscosity of PUR systems containing HS fillers is increased, and the expansion of the cells is reduced. Because of this, the HS fillers react with isocyanate groups, forming an interpenetrating cross-linked network, which in turn disturbs the gas release, reducing the size of cells.

The cellular structure affects the apparent density of PUR foams (Table 4). Due to the incorporation of HS, HS/SO, and HS/TO, the value of apparent density increases by 6, 16, and 17%, respectively. A greater apparent density of PUR foams containing HS fillers should be attributed to the increased viscosity and limited expansion of modified PUR systems. Moreover, the apparent density of modified PUR foams is further enhanced by the molecular weight of HS fillers.

**Figure 5:**
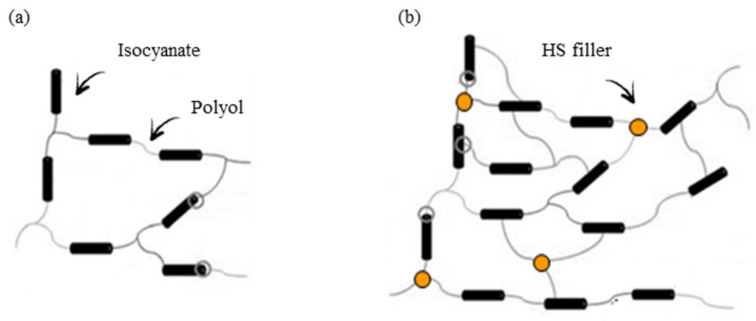
Crosslinking structure of (**a**) neat PUR foams and (**b**) PUR foams containing HS fillers.

Thermal conductivity (λ) is an important parameter that defines the thermal insulation properties of PUR foams [10]. The value of λ measured for neat PUR_0 is 0.025 W m^−1^ K^−1^ (Table 4). The addition of HS filler has no significant influence on the value of λ; however, the incorporation of impregnated HS/SO and HS/TO increases the value of λ by about 20 and 24%, respectively. In general, the value of λ involves the thermal conductivity of the gas captured in the foam cells (λ_gas_), solid backbone of the foams (λ_solid_), heat transfer between foam cells (λ_solid_), and gas convection (λ_convection_). With the addition of HS fillers, a greater number of filler particles are built in the polymer matrix, thus the value of λ_solid_ increases. As mentioned previously, due to the increased viscosity of modified PUR foams, the functional groups of filler particles are involved in the reaction with isocyanate groups. This results in an increased crosslinking degree (Figure 5) of PUR molecular chains and formation of PUR foams with smaller cells and a greater apparent density, which additionally increases the value of λ_solid_. Besides this, all series of PUR foams are in line with commercial requirements for commercial thermal insulation boards [49]. 

Another important parameter determining the further use of PUR foams as construction materials is water uptake. Previous studies reported that the cellular structure of PUR foams and the hydrophobic nature of the filler affects the water uptake of porous materials [11,50,51]. The results of the water uptake of PUR foams containing HS fillers are presented in Table 4. When compared with neat PUR_0, the addition of HS filler increases the water uptake of PUR_HS—the value increases from 21.5 to 23.8%. Increased absorption of water may be connected with a less uniform structure of PUR_HS and a greater number of open cells. Moreover, the particles of HS filler tend to agglomerate, creating a “pathway” that facilitates water penetration into the foam structure [11,50,51,52]. Surprisingly, an opposite effect has been observed for PUR foams containing impregnated HS/SO and HS/TO—the water uptake slightly decreases by ~6% due to the hydrophobic character of impregnated RC fillers, which was also confirmed by a decreased value of the contact angle (*θ*) (Table 4).

### 3.4. Mechanical Characteristics of PUR Foams

**Figure 6:**
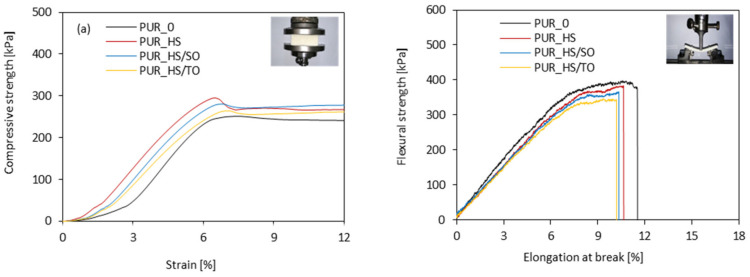
Mechanical behavior of PUR foams’ (**a**) compressive and (**b**) flexural behavior.

The mechanical properties of porous materials mostly depend on their cellular structure [11]. The compressive strength–strain graph measured during the external loading is presented in Figure 6a. All samples are characterized by analog plots—the linear region refers to the elastic response of PUR foams, while the second region (which presents a plateau region) refers to the plastic deformation and cell’s rupture. When compared with neat PUR_0, in the case of foams containing HS fillers, the transition from elastic to plateau region is more abrupt, while the elongation at break decreases, indicating the more rigid structure of modified foams. A similar trend has been found in previous studies as well [53,54].

**Figure 7:**
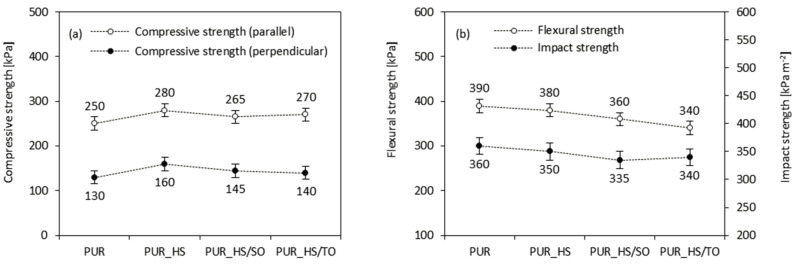
Mechanical characteristics of PUR foams containing HS fillers: (**a**) compressive strength, (**b**) flexural and impact strength.

Depending on the type of HS filler, the compression strength increases by ~12, ~6, and ~8% for PUR_HS, PUR_HS/SO, and PUR_HS/TO, respectively (Figure 7a). Previous studies have shown that the value of compression strength depends on the crosslinking density of PUR foams, which refers to the content of hard segments (urethane groups) [55,56]. It may be concluded that the incorporation of HS fillers affects the crosslinking density of PUR foams. After the incorporation of cellulosic HS fillers, active groups of HS (i.e., hydroxyl groups of cellulose and lignin) are involved in the reaction with isocyanate groups, increasing the number of urethane groups and creating a more dense structure of PUR foams. An increased number of urethane groups provides additional crosslinking points, increasing the number of hard segments and enhancing the mechanical performances of PUR foams.

A different trend is observed in the case of measuring the flexural and impact strength (Figure 7b). The addition of HS fillers affects both parameters, decreasing their values. Depending on the HS filler type, the value of flexural strength decreases by 3–13%, while the value of impact strength decreases by 3–7%. The higher content of hard segments makes the PUR foams harder but more brittle. Thus, the values of flexural and impact strength decrease when the HS fillers are added. Besides, some aggregates of HS particles, which are localized in the PUR structure, can act as stress concentrations, promoting the cracking of the samples and deteriorating the mechanical performances of PUR foams. As presented on the stress–elongation graph (Figure 6b), all modified PUR foams exhibit a similar mechanical performance, which involves elastic and plastic deformation; however, the value of elongation at break decreases when the HS fillers are added. The aggregates of HS particles act as additional defects, promoting the cracking of the sample under an external force.

### 3.5. Dynamic–Mechanical Properties of PUR Foams

**Figure 8:**
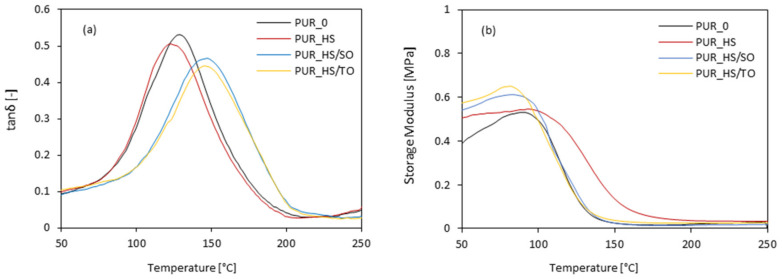
The dynamic–mechanical properties of PUR foams containing HS fillers: (**a**)—tanδ and (**b**)—storage modulus results.

The dynamic–mechanical properties of PUR foams are presented in Figure 8. The modification of PUR foams with HS fillers affects the glass transition temperature (T_g_), determined as a maximum peak of the curve loss tangent (tanδ) in the function of the temperature. When compared with neat PUR_0, the addition of HS fillers increases the value of T_g_. Among all series of PUR foams, the highest value of T_g_ is observed for PUR foams containing impregnated HS fillers—the value of T_g_ increases from 128 to 148 °C for PUR_HS/SO and PUR_HS/TO. The results confirm that the addition of impregnated HS fillers results in the formation of PUR foams with a greater cross-linking density, limiting the mobility of polymer chains. Besides, HS filler particles that are built into the foam structure can act as additional blockages, increasing the amount of energy that is required to achieve the T_g_. Moreover, when compared to neat PUR_0, the addition of each type of HS filler results in the improvement of the storage modulus of PUR foams. The greatest improvement is observed for PUR foams containing impregnated HS fillers. This may be connected with an increased viscosity of PUR systems containing HS/SO and HS/TO, which limits the mobility of polymer chains, leading to the formation of PUR foams with higher stiffness. In addition, the filler particles can act as reinforcing centers, effectively transferring the external stresses from particles to the PUR matrix. Similar results have been reported in previous works as well [57,58].

### 3.6. Thermogravimetric Analysis (TGA) of PUR Foams

**Figure 9:**
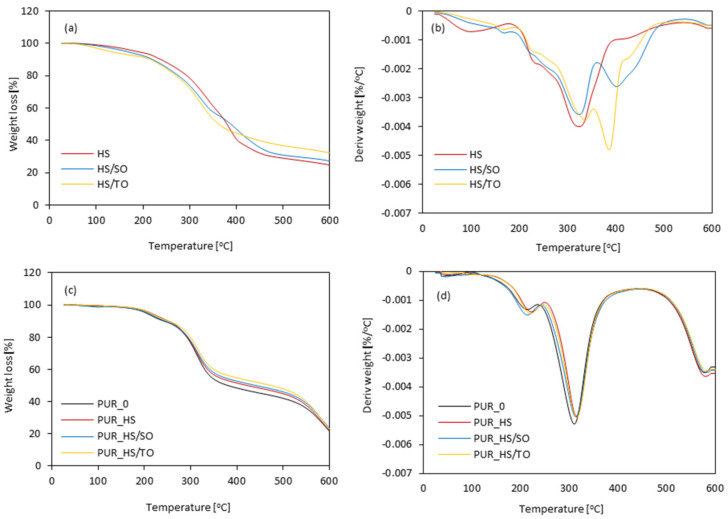
TG and DTG results obtained for (**a**,**b**) HS fillers, and (**c**,**d**) PUR foams.

Figure 9a,b present the TG/DTG curves of HS fillers. The first loss of mass occurs at ~100 °C and refers to the release of water accommodated in the fillers. A second loss occurs in the range of 300–400 °C and is mostly connected with the decomposition of the cellulosic derivatives—cellulose and lignin, respectively [34,59]. Similar TG curves have been observed in the case of other cellulosic fillers [60].

Figure 9c,d present TG and DTG curves of PUR foams. The resulting data are depicted in Table 5. Only slight shifts in the level of each curve are observed, which correspond to the weight loss. However, a meaningful change in the position of the main peak connected with the decomposition temperature of PUR can be observed. When compared with neat PUR_0, the addition of HS fillers results in the production of PUR foams with higher values of T_2%_, which refers to the release of volatile products presented in the HS fillers. Previous studies have reported that cellulosic fillers tend to degrade in the range of lower temperatures, thus a higher value of T_2%_ should be attributed to the partial crosslinking between functional groups of HS fillers and isocyanate groups [61]. The second degradation of mass loss occurs in the range of 309–326 °C and refers to the thermal decomposition of hard segments—urethane bonds [62,63]. A slower degradation of mass is observed when HS fillers are added and confirms a higher crosslinking degree of modified PUR foams. The third stage, which occurs at nearly 590 °C, refers to the degradation of cellulose and lignin. The thermal degradation mechanism of PUR foams modified with HS fillers seems similar to PUR_0. The addition of HS fillers results in a slight increase in mass loss of PUR foams, because of the presence of lignocellulosic compounds and incomplete miscibility of soft and hard segments of PUR [64]. Moreover, the rigid structure of cellulosic HS fillers can cause them to act as additional cross-linker centers between PUR matrices, improving the heat resistance of modified PUR foams—the value of mass residue at 600 °C increases from 23.8% (for PUR_0) to 26.2 and 26.5% for PUR_HS/SO and PUR_HS/TO, respectively. An analog trend has been shown previously in the case of PUR foams containing another type of cellulosic filler [65,66]. For example, Tian et al. [61] have stated that the reduced thermal decomposition PUR foams enhanced with soy-protein filler refer to the higher degree of crosslinking of PUR foams, due to the reaction between functional groups of soy-protein and PUR systems. More cross-linked structure of PUR foams limits the number of volatile products that are generated and released during the thermal degradation process, reducing the thermal decomposition of PUR foams. A similar explanation may be found in our study as well.

### 3.7. Flammability of PUR Foams

**Figure 10:**
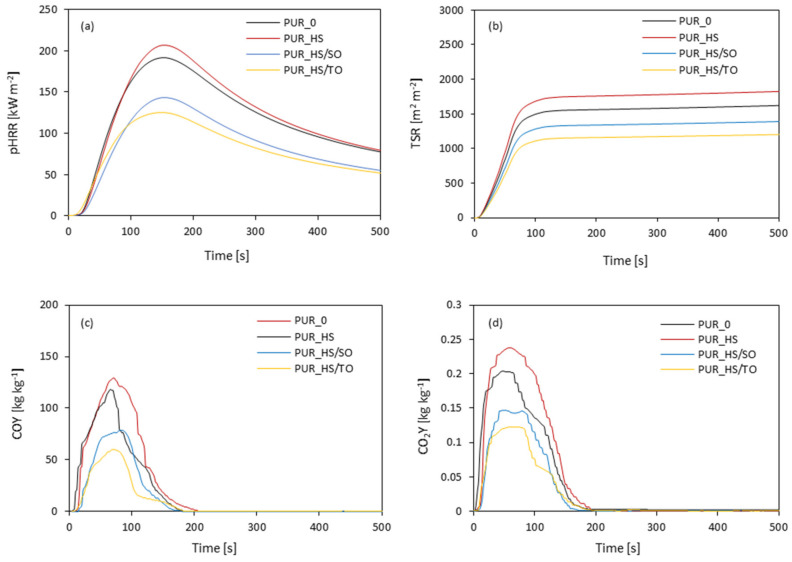
Cone calorimeter results: (**a**) pHRR, (**b**) TSR, (**c**) COY and (**d**) CO_2_Y.

The addition of RC fillers significantly affects the flame retardancy of PUR foams. The results of the cone calorimeter test are presented in Figure 10 and Table 6.

When compared to neat PUR_0, the addition of HS fillers increases the ignition time (IT) and this trend is more prominent in the case of PUR foams containing impregnated HS/SO and HS/TO fillers—the value of IT increases from 3 s to 5 and 6 s, respectively. The results presented in Figure 10a indicate that the value of heat peak release (pHRR) increases by ~8% when the non-treated HS filler is added. An opposite effect is observed for PUR foams containing impregnated RC fillers—the value of pHRR decreases by ~30 and 20% for PUR foams containing HS/SO and HS/TO, respectively. Such an improvement may be connected with the formation of a char layer, which effectively reduces the release of combustible gases. In general, the pHRR values of modified PUR foams are in the line with the regulations, which determines the accessible value of pHRR as 300 kW m^−2^ [67]. Below this value, the materials are approved for use as insulating materials for building construction. Moreover, the addition of impregnated HS fillers decreases the value of total smoke release (TSR) (Figure 10b). Compared to neat PUR_0, the value of TSR decreases by 15 and 27% for PUR_HS/SO and PUR_HS/TO, respectively. Moreover, due to the incorporation of HS fillers, the values of COY and CO_2_Y slightly decrease (Figure 10c,d). This may be connected with the fact that HS filler particles act as a physical barrier for flame spread, reducing the heat transfer through the PUR sample and decreasing the intensity of the flame.

**Figure 11:**
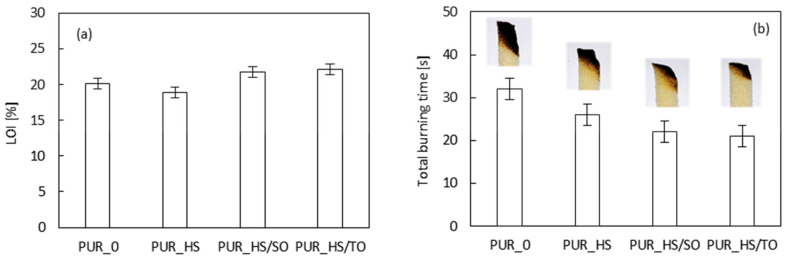
(**a**) LOI and (**b**) total burning time of PUR foams.

The results of limiting oxygen index (LOI) are presented in Figure 11a. Similarly to the results of the cone calorimeter test (pHRR and TSR), the addition of non-treated HS filler decreases the LOI value from 20.2 to 19.8%. Increased value of LOI is observed for PUR foams containing impregnated HS fillers—the LOI increases to 20.8 and 21.6%, for PUR_HS/SO and PUR_HS/TO, respectively. Chan et al. [68] have reported similar results in the case of PUR foams enhanced with ramie fibers in the amount of 0.2–0.8 wt.%. It has been shown that the carbonization of the fibers during the combustion process results in the formation of pores, preventing the propagation of the flame through the PUR matrix and increasing the value of LOI.

The self-extinguish capacity was measured according to the UL-94 standard, which corresponds to the burning time measured after continuous ignitions of the samples. According to the UL-94 standard, neat PUR_0 was not classified under test standard, while PUR foam containing RC fillers achieved a V-0 rating. Moreover, the total time of burning was evaluated. According to the results presented in Figure 11b, the flame keeps longer in the case of neat PUR_0, for which the total time of burning was 32 s. The total time of burning decreases when the HS fillers were added. The most visible effect is observed in the case of PUR foams containing impregnated HS fillers—the value decreases to 22 and 21 s for PUR_HS/SO and PUR_HS/TO, respectively. The results of UL-94 are in agreement with the results of LOI. In summary, the highest standards among UL-94 and LOI tests are obtained for PUR foams containing impregnated HS fillers.

## 4. Conclusions

PUR foams were reinforced with 2 wt.% non-treated and impregnated HS fillers were successfully synthesized. It has been shown that each type of HS filler affects the morphology and further mechanical, thermal, and insulating properties of PUR foams. It has been shown that the addition of HS fillers improved the mechanical characteristics of PUR foams. Among all modified series, the greatest improvement was observed after the incorporation of non-treated HS filler—when compared with neat foams, the value of compressive strength increased by ~13%. Moreover, the incorporation of impregnated HS fillers resulted in the improvement of thermal stability and flame retardancy of PUR foams. For example, the addition of both types of impregnated HS fillers significantly decreased the value of heat peak release (pHRR), total smoke release (TSR), and limiting oxygen index (LOI). Moreover, the PUR foams containing impregnated fillers were characterized by improved hydrophobicity and limited water uptake. The obtained results confirmed that the modification of PUR foams with non-treated and impregnated HS fillers may be a successful approach in producing polymeric composites with improved properties.

## Figures and Tables

**Table 1:** Recent works on filler reinforced polyurethane foams—effect of different fillers on mechanical properties of polyurethane foams.

Filler Used	Percentage of Filler	Results
Kenaf fibre	20–50 wt.%	Improvement of mechanical properties [12]
Pulp fibre	0–5 wt.%	Deterioration of mechanical properties, improvement of thermal stability [13]
Rice husk ash	0–5 wt.%	Improvement of mechanical properties and flame-retardancy, deterioration of thermal conductivity [14]
Cellulose microfibres	0–2 wt.%	Improvement of mechanical properties [15]
Cellulose nanocrystals	1–8 wt.%	Improvement of mechanical properties [16]
Egg shell waste	20 wt.%	Improvement of mechanical properties and thermal stability [17]
Potato protein waste	0.1–5 wt.%	Deterioration of mechanical properties and thermal stability with increasing filler content [18]
Buffing dust waste	0.1–5 wt.%	Deterioration of mechanical properties and thermal stability with increasing filler content [19]
Keratin feathers	0.1–1.5 wt.%	Mechanical properties and thermal stability decrease with increasing filler content [20]
Forest based wastes	10 wt.%	Deterioration of mechanical properties and thermal conductivity, improvement of flame-retardancy [11]
Ground coffee	2.5–15 wt.%	No significant influence on the mechanical and thermal properties, reduced brittleness and aging process [21]
Jute fibre	0.5–4 wt.%	Deterioration of mechanical properties [22]
Ramie fiber	0.2–0.8 wt.%	Improvement of mechanical properties, thermal stability, and flame-retardancy [23]
Rapeseed cake	30–60 wt.%	Improvement of mechanical properties, thermal stability, and flame-retardancy [8]
Wood flour	0–15 wt.%	Deterioration of mechanical properties, improvement of thermal conductivity and thermal stability [24]
Coir fibre	2.5 wt.%	Improvement of mechanical properties [25]
Fly ashes	5–35 wt.%	Improvement of mechanical properties and fire resistance [26]
Cinnamon extract, green coffee extract, cocoa extract	10 wt.%	Improvement of susceptibility to biodegradation [27]
Soy protein	2.4–9.6 wt.%	Improvement of mechanical properties, deterioration of thermal stability [28]

**Table 2:** Composition of PUR composite foams.

Component	PUR_0	PUR_HS	PUR_HS/SO	PUR_HS/TO
	Parts by Weight (wt.%)			
STEPANPOL PS-2352	100	100	100	100
PUROCYN B	160	160	160	160
Kosmos 75	6	6	6	6
Kosmos 33	0.8	0.8	0.8	0.8
Tegostab B8513	2.5	2.5	2.5	2.5
Water	0.5	0.5	0.5	0.5
Pentane/cyclopentane	11	11	11	11
Hemp shives (HS)	0	2	0	0
Hemp shives/sunflower oil (HS/SO)	0	0	2	0
Hemp shives/tung oil (HS/TO)	0	0	0	2

**Table 3:** The impact of HS fillers on viscosity and processing times of PUR systems.

	Dynamic Viscosity *η* (mPa·s)			Processing Times (s)		
	0.5 RPM	50 RPM	100 RPM	Cream Time (s)	Free Rise Time (s)	Tack-Free Time (s)
PUR	850 ± 10	410 ± 8	330 ± 9	42 ± 4	280 ± 9	350 ± 12
PUR_HS	1100 ± 10	980 ± 10	420 ± 12	50 ± 2	320 ± 9	345 ± 10
PUR_HS/SO	1800 ± 11	1300 ± 10	750 ± 10	60 ± 1	355 ± 8	330 ± 8
PUR_HS/TO	2200 ± 11	1550 ± 10	850 ± 10	66 ± 2	370 ± 8	320 ± 8

**Table 4:** Selected properties of PUR foams containing HS fillers.

Sample	Closed-Cell Content (%)	APPARENT Density (kg m^−3^)	Thermal Conductivity (W m^−1^ K^−1^)	Water Uptake (%)	Contact Angle (°)
PUR_0	91.4 ± 0.5	37.2 ± 0.6	0.025 ± 0.001	21.5 ± 0.6	123 ± 1
PUR_HS	89.2 ± 0.4	40.6 ± 0.7	0.026 ± 0.001	23.8 ± 0.5	120 ± 1
PUR_HS/SO	88.6 ± 0.4	43.1 ± 0.6	0.030 ± 0.001	19.2 ± 0.6	129 ± 1
PUR_HS/TO	85.6 ± 0.4	43.5 ± 0.6	0.031 ± 0.001	20.1 ± 0.5	130 ± 1

**Table 5:** The results of TGA and DTG analysis of PUR foams.

Sample	T_max_ (°C)			Residue at 600 °C (wt.%)
	1st Stage	2nd Stage	3rd Stage	
PUR_0	218	309	581	23.8
PUR_HS	217	318	584	23.1
PUR_HS/SO	220	325	586	26.2
PUR_HS/TO	222	326	589	26.5

**Table 6:** Flammability parameter measured for PUR foams.

	IT [s]	pHRR [kW m^−2^]	THR [MJ m^−2^]	TSR [m^2^ m^−2^]	COY [kg kg^−1^]	CO_2_Y [kg kg^−1^]
PUR_0	4	268	21.0	1550	0.204	0.204
PUR_HS	3	288	22.5	1740	0.238	0.238
PUR_HS/SO	5	172	20.2	1315	0.147	0.147
PUR_HS/TO	6	211	19.4	1135	0.122	0.122
